# Optical Thickness Monitoring as a Strategic Element for the Development of SPR Sensing Applications

**DOI:** 10.3390/s20071807

**Published:** 2020-03-25

**Authors:** Donato Luna-Moreno, Araceli Sánchez-Álvarez, Melissa Rodríguez-Delgado

**Affiliations:** 1 Centro de Investigaciones en Óptica AC, Div. de Fotónica, Loma del Bosque 115, Col. Lomas del Campestre C.P. 37150, León, Gto, Mexico; dluna@cio.mx; 2 Universidad Tecnológica de León, Electromecánica Industrial, Blvd. Universidad Tecnológica #225, Col. San Carlos C.P. 37670, León, Gto, Mexico; asalvarez@utleon.edu.mx; 3 Universidad Autónoma de Nuevo León, Facultad de Ciencias Químicas. Av. Universidad S/N Ciudad Universitaria, San Nicolás de los Garza C.P. 66455, Nuevo León, Mexico; 4 Centro de Investigación en Biotecnología y Nanotecnología (CIByN), Facultad de Ciencias Químicas, Universidad Autónoma de Nuevo León. Parque de Investigación e Innovación Tecnológica, Km. 10 autopista al Aeropuerto Internacional Mariano Escobedo, Apodaca 66629, Nuevo León, Mexico

**Keywords:** surface plasmon resonance, optical constant, thin-film coating, optical properties of surfaces, biosensor

## Abstract

The importance of the monitoring of thickness and rate deposition is indispensable for the fabrication of thin film sensors, such as SPR sensors. The sensitivity of SPR responses varies with the thickness of the film, as well as the linear range. Thus, in the present work, we presented an experimental study of the plasmonic response of Cr/Au thin films deposited onto glass slides by evaporation, based on both a rotation and no-rotation system. The results show that the thickness of the gold film varies from 240 to 620 Å, depending on the glass slide position. The SPR response curves obtained experimentally were compared with simulated plasmonic responses and different parameters such as resonance angle, and the depth, slope and half-width of the SPR curve were analysed.

## 1. Introduction

The surface plasmon resonance (SPR) technique has become an active field of research due to the constant development of devices capable of detecting chemical and biological substances for a broad range of applications, from biomedical to food industries. SPR devices are based on the interaction of the electromagnetic excitations of surface plasmons (charge density oscillations) at an interface between a thin metallic layer (~50 nm of Au or Ag) and a dielectric medium [1]. Thus, this phenomenon highly depends on the thickness of the multilayer structure, and any factor that causes variations in it could generate significant changes in the plasmon dispersion, altering the SPR curve. The analysis of the SPR response curve provides specific information related to sensing applications. For example, from the minimum point in the curve is obtained the maximal phase variation with refractive indices of the adjacent medium, whereas the resonance slopes indicate the amplitude changes and thus, the sensitivity of the device. Furthermore, the width of the curve gives information about effects related to the absorption process in the metal layer [2,3].

In this sense, the effect of deposition parameters employed during the formation of the sensing thin films plays a critical role in the SPR sensors’ performance, with the thickness and surface morphology being especially important [4,5]. Several parameters for the deposition of metallic materials employed in SPR have been studied by McPeak et al., including the pressure of the vacuum chamber, the rate of evaporation, the substrate temperature, and the thickness of the thin film [6]. In particular, factors related to the rate of evaporation cause significant effects on the roughness of thin films, since the condensation of evaporated material onto a substrate produces isolated islands of metal, causing the uniformity of the layer thickness [7]. Furthermore, the resonance angle gradually decreases when the roughness of the gold film varies from rough to smooth, showing discrepancies in the angle obtained by theoretic calculations in comparison with experimental data [8]. In order to achieve specified thickness profiles, different schemes of evaporation [9] and the use of shadowing masks have been tested, in both spherical and aspherical substrates [10,11]. For example, a simple rotating system based on the rotation of the substrate allows the thickness deposited at any point of the substrate to be similar to the average of the mass deposited. However, in this scheme, the sensitivity of the thickness monitor diminishes with the increasing build-up of mass [12], causing errors in thickness estimation. Thus, it is essential to establish methods that allow the proper characterization of thin films.

Depending on the properties needed in a thin film for a specific sensing application by SPR, different mechanical elements can be adapted within the evaporation system, allowing different deposition configurations. Previous works have already reported the adaptations of different mechanisms during a deposition process, e.g., the evaporation of gold or silver in an optical fibre tapered over a section [13] or around the fibre using manual [14] or electronic rotation systems [15,16,17].

Thus, the present work established the characterization of Cr-Au thin films deposited onto glass slides (BK7) by evaporation based on both a rotation and no-rotation system. The obtained substrates were evaluated by SPR measurements to obtain their sensing properties. The SPR response curves obtained experimentally were compared with simulated plasmonic responses, and further analyzed to extract different parameters such as resonance angle, and the depth, slope and half-width of the curves. Finally, according to the experimental data obtained by the different thin films, different sensing applications were suggested.

## 2. Materials and Methods

### 2.1. Theoretical Simulation Using the Fresnel Equations and the Matrix Method

There are several simulation models for surface plasmon resonance, such as the vector method, where all the reflections are coherently added across each layer in a multilayer system. On the other hand, the matrix method is widely employed in optical thin film design due to its ease of mathematical expression and easy understanding of the physical phenomenon.

In our work, a theoretical model was established based on the Fresnel equations and the matrix formalism to describe the post-selected SPR sensing system as a multilayer thin film assembly [18].

The theoretical reflectance of a multilayer thin film was expressed by $R=rr^{*}$, where *r* is the amplitude reflectance calculated by the expression

(1) $$r=\frac{\eta_{0}-Y}{\eta_{0}+Y}$$

Here, *η*_0_ is the admittance of the interface of the incident medium, and *Y* is the admittance of the multilayer system, and is given by the characteristic matrix

(2) $$Y=\frac{H}{E}$$

where *H* and *E* represent the amplitude of the magnetic and electric field tangential component, respectively. These components are expressed in the matrix representation

(3) $$\left[\begin{array}{c} E\\ H \end{array}\right]=\prod_{j=1}^{k}\left[\begin{array}{cc} \cos\delta_{j} & \frac{i\sin\delta_{j}}{\eta_{j}}\\ i\eta_{j}\sin\delta_{j} & \cos\delta_{j} \end{array}\right]\left[\begin{array}{c} 1\\ \eta_{s} \end{array}\right]$$

where optical admittance of the jth layer film is *η_j_* = *n_j_/cosθ_j_* for transversal magnetic (TM) polarization, and *η_j_* = *n_j_cosθ_j_* for TE (transversal electric) polarization. *δ_j_* is the phase thickness (given for *δ_j_ = 2δπ*n*_j_d_j_(cosθ_j_)/λ*, *d* is the physical thickness, *n* is the refractive index of the thin film, *λ* is the wavelength, *η_s_* is the relative admittance of the substrate or sample medium, and *ϴ* is the angle for the incident medium). The refractive index is an optical parameter, crucial in a sample, which can be defined in terms of real and imaginary elements.

The electric and magnetic fields are expressed as column vectors, and each film as a transfer matrix. The calculus involves successive multiplications of the column vectors by the transfer matrix. The system is then reduced to finding the reflectance of the pure ideal interface between an incident medium *η*_0_ and a medium of admittance *Y*.

### 2.2. Evaporation of Cr/Au Thin Films

Cr/Au films were deposited onto glass slides and hemicylindrical shaped prisms, fabricated in our facilities using BK7 glass (refractive index of 1.51509 for λ = 632.8 nm [19]). The deposition process occurred by evaporation with and without a rotation system. Substrates (22 x 22 mm) were placed in the vacuum chamber (High Vacuum Coating Plant BA510, Balzers High Vacuum Corp., Santa Ana, Calif., USA), which combines an electron gun and thermal evaporation. Chrome with a purity of 99.999 % (Kurt J. Lesker Co., Clairton, PA, USA) was evaporated by electron gun evaporation with a rate of 1.0 Å s^−1^, in an atmosphere of 8 × 10^−6^ mbar. Gold with a purity of 99.999 % (pellets of 1/8’’ of diameter, part # EVMAUXX50G, Kurt J. Lesker Co., Clairton, PA, USA) was evaporated at a rate of 5 Å s^−1^ in an atmosphere of 8 x 10^-6^ mbar by thermal evaporation. The thickness of the deposited chrome and gold films was estimated using a quartz crystal thickness monitor (XTC/2 Depositions Controllers Leybold Inficon quartz monitor, San Jose, CA, USA).

### 2.3. SPR Measurements

The SPR setup was based on a homemade platform, previously reported in [20], consisting of two motorized rotating plates where the prism (Kretschmann configuration) is placed on the base of the superior plate, and a photodetector system is mounted on the lower plate. A fluidic system was coupled, consisting of a Teflon cell with an inlet and outlet tube through which solutions were continuously run by use of a syringe pump at a fixed flow rate [20]. Then, two sensing applications, employing the thin films obtained from the different evaporation schemes, were performed based on SPR measurements at a fixed angle. The first sensing application reported in [21] was the detection of different alcohol content ranging from 35 to 46%v/v, where 1 mL of isopropyl alcohol was evaporated and delivered over the Au film, then measured by SPR at 43.6° to later be compared with the signal obtained in air. As a second application, reported in [22], the biofunctionalization of a gold film was evaluated in real-time by SPR, employing the EDC/NHS method for the immobilization of a polyclonal antibody (50 µg mL^−1^) onto the gold surface. The obtained signals from the antibody-antigen effect were directly proportional to the concentration of antigen (39.1-122 µg mL^−1^) in the samples.

## 3. Results and Discussions

Film deposition was performed in a vacuum chamber with both an e-beam and a thermal vapour deposition system. The transduction surface in SPR devices is usually a thin gold film (~ 50 nm) on a prism or a glass slide (optically coupled to a prism through a refractive-index-matching oil). Although the thin film coating on a glass slide is the most practical, the metal film provokes a problem due to the interference of the light beam reflected off the prism base and beams reflected at the interface of the oil and glass, if they are imperfectly matched. A total internal reflection at the prism-oil interface occurs, and the light does not reach the gold film anymore. As a consequence, data for θ > 80 ° are to be interpreted with great care; data for θ > 85 ° are useless. However, if the measurement range is around 80 degrees, this can be solved using a prism with a higher refractive index. This means that the lower the refractive index of the prism, the higher the sensitivity of the SPR system, but the lower the measurement range of refractive index changes would be. Another possible strategy to avoid this effect is to evaporate the metal directly on the prism base. Since gold has a very poor adherence to glass, a layer of chromium or titanium dioxide needs to be deposited between the glass and the gold [23]. However, the thin film of Cr or TiO_2_ is very difficult to remove unless a grinding process is employed, unlike the gold or silver films that can be removed with a quick polishing process.

### 3.1. Characterization of the Gold and Chrome Thin Film

**Figure 1:**
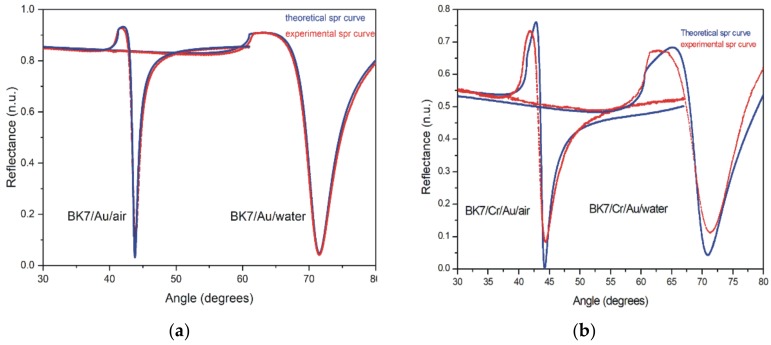
Theoretical (blue line) and experimental (red line) normalized SPR curve, employed to estimate the complex refractive index and thickness of each thin film. The measurements were performed in air and water for (**a**) prism BK7 covered with Au film and (**b**) prism BK7 covered with Cr/Au.

The thin film’s characterization plays a crucial role in the sensing properties, since the adequate control of the optical parameters guarantees a better performance of the sensor. These parameters are responsible for the refractive indexes range that the sensor will be capable of detecting, as well as the resolution in which those changes can be detected. Gold thin films are the most common transducer employed in SPR biosensing, mainly due to its chemical stability and its straightforward chemical functionalization protocols. However, its adhesion to the prism is very low, so it is necessary to use a layer of chromium (or titanium) as an adherent between them. The refractive index of thin films strongly depends on the deposition technique, yielding a different surface morphology response [24]. In this study, gold thin films were deposited on a BK7 prism by thermal evaporation. SPR measurements were performed to characterize the optical response of the gold thin films. The evaluation was recorded by an angular sweep using a polarized He-Ne laser, (Uniphase mod. 1101P), centered at 632.8 nm of 1.5 mW of minimum-output power and a minimum polarization ratio of 500:1, which was launched into a hemicylindrical shaped prism with its flat face looking downward to validate normal incidence, a dielectric medium air (n = 1.0000) and distilled water at 25 °C (n = 1.3328) [25]. Subsequently, the experimental data obtained from the SPR curve was theoretically fitted using the square least method [3]. The parameters obtained for the gold film were a thickness of d = 500 Å, and a refractive index of N = 0.1758 - 3.3895i (see Figure 1a).

In addition, a different substrate with Cr/Au films was prepared. A chrome layer of approximately 30 Å was deposited by e-gun evaporation, and a gold thin film of 450 Å thickness was placed by thermal evaporation. The change in thickness from 500 Å to 450 Å for the Au thin film is for the widening of the SPR curve due to the adhesion of the Cr thin film; that is, the Cr layer is as thin as possible to avoid effects in the SPR curve of the Au. Then, the SPR signal with angular interrogation was measured to determine the characteristics of the chrome film using the previously obtained gold film parameters. In this case, the values for Cr thin film were d = 28 Å and N = 3.3101-4.2733i, using air and distilled water as the dielectric (see Figure 1b). In this process, a refractive index of N = 0.1758 – 3.3895i and 435 Å of thickness was obtained for the gold thin film.

### 3.2. Effect of the Intensity of the Incident Light in the SPR System

**Figure 2:**
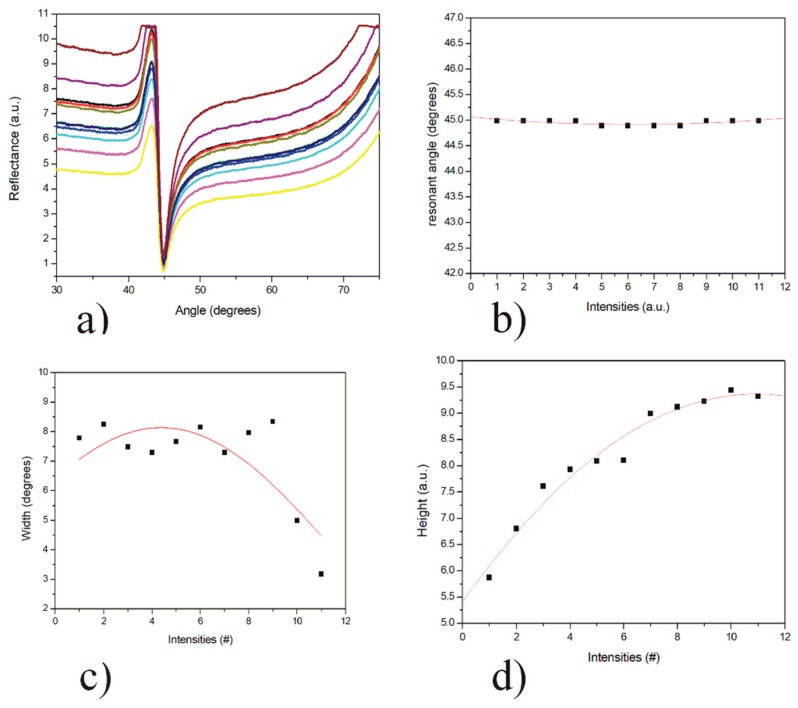
Analysis of the influence of the intensity of the illumination source. (**a**) Experimental SPR curves of a substrate with a Cr/Au thin film, (**b**) resonant angle for the SPR curves at different intensities, (**c**) graph of the width of the SPR curves, (**d**) graph of the height of the SPR curves.

One more critical factor in SPR measurements is the photodetector responsivity, which depends on the intensity of the light. If the intensity of the incident light on the surface of the photodetector is very high, the photodetector signal will be saturated. However, a very low light intensity may create a flat SPR curve. Therefore, the ideal incident light intensity is one that takes into account the value of the photodetector in the area near the critical angle without a light saturation. The incident intensity of the light is regulated using a neutral variable filter. These filters are usually made with chromium or chromium/nickel alloys to attenuate the light intensity without altering its polarization. Usually, the resonance angle will be the same for the SPR measurements at different intensities of incident light (see Figure 2a,b). However, the width (Figure 2c) and height (Figure 2d) of the response curves will be significantly affected, especially the height of the curve (as the intensity of the incident light increases it will cause an increase in the height of the SPR curve). This is essential to consider since, for biosensing measurements, the prism must be placed at the angle of half of the curve slope (highest sensitivity); that is, at half the height of the SPR curve.

### 3.3. Evaporation of Cr/Au with no-Rotation System

**Figure 3:**
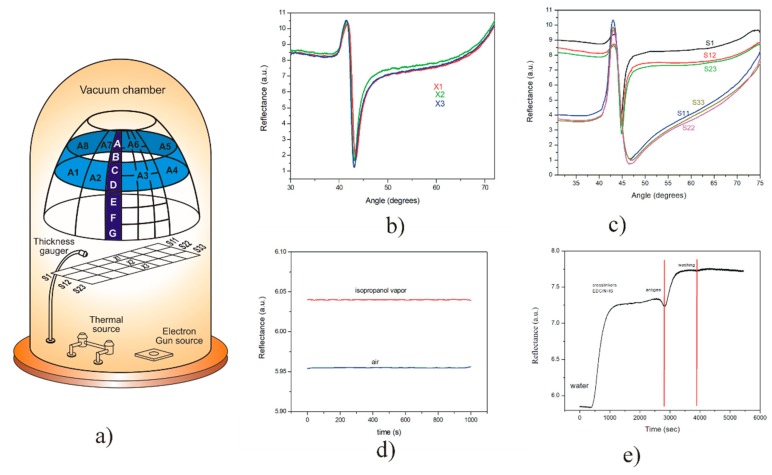
(**a**) Schematic representation of the location of substrates during deposition of Cr/Au with a rotation system (the upper cone) and without rotation (close to the thickness gauger in a vacuum chamber); (**b**) SPR curves of substrates X1, X2 and X3 that correspond to the central position of coated substrates without rotation (quartz monitor close to position X1), (**c**) SPR curves of the three substrates of the extreme left and extreme right in the system; (**d**) SPR measurement of the presence of isopropyl alcohol vapours in comparison with air, using a Cr/Au substrate of thickness 28/300 Å, based on measurements of intensity changes by SPR at a fixed angle; (**e**) SPR signal of the biofunctionalization of Cr/Au thin film corresponding to substrate X1, employing the EDC/NHS method for polyclonal antibody (50 µg mL−1) immobilization for an antigen-antibody recognition.

Cr/Au thin films were deposited onto glass slides (BK7) by evaporation without rotation. Figure 3a shows the position of the substrates during the evaporation. The substrates X1, X2, X3, corresponds to substrates in the centre row; S11, S22, S33 in the far left; and S1, S12, S23 in the right. The thickness measured in the monitor was 450 Å and 30 Å for the Au and Cr films, respectively. From the fitting analysis of the SPR curves, and using the complex refractive index values of N_Au_ = 0.1758 - 3.3895i and N_Cr_ = 3.5652 - 4.362i for the Au and Cr thin films, respectively, we find the values of the thickness of the substrates mentioned above to know the distribution of uniformity of thicknesses. Meanwhile, the value estimated by adjusting the theoretical/experimental SPR curves of X1 (intermediate substrate) was 454 Å and 28.7 Å for the Au and Cr thin films, respectively. Figure 3b shows the SPR curves corresponding to the substrate X1 and the two neighboring substrates (X2, X3), which comprise the three central substrates of each deposition row (Figure 3a). On the other hand, the substrates located farthest from the quartz monitor showed a notable difference in their thickness value. The far-left substrates (S11, S22, S33) exhibited an approximate thickness of 27 Å of Cr and 617 Å of Au, while the far-right substrates displayed an estimated thickness of 24 Å of Cr and 238 Å of Au. Figure 3c shows the effect that thickness has on the SPR curves of substrates designated according to the distance from the evaporation source, the substrates closest to the quartz monitor being those with the most accurate thickness. Thus, it is essential to highlight that within a distance of 35 cm (rack with 11 substrates of 22 × 2 mm) the thickness of the gold film varies from 240 to 620 Å. Despite the difference in thicknesses, the substrates with a lower amount of gold can be used for gas sensing at fixed-angle. Figure 3d shows the measurement of the presence of isopropyl alcohol vapours in comparison with air, using a Cr/Au substrate of thickness 28/300 Å. The method was based on measurements of intensity changes by SPR at a fixed angle [21]. Although this Cr/Au thin film resulted in being considerably thin and far from the expected thicknesses, the film showed a very wide SPR curve, and it has a considerable height, which it is possible to use for the detection of air-like volatile elements, as shown in Figure 3d. However, the thin film would have a low response for SPR angular scan measurements due to a full width at the maximum half (FWHM).

On the other hand, ideal films like the one obtained in substrate X1 are strong candidates to be employed in biosensing applications. Figure 3e shows the use of this film in the biofunctionalization of the thin gold layer, performed in a recent work [22].

### 3.4. Evaporation of Cr/Au with Rotation

**Figure 4:**
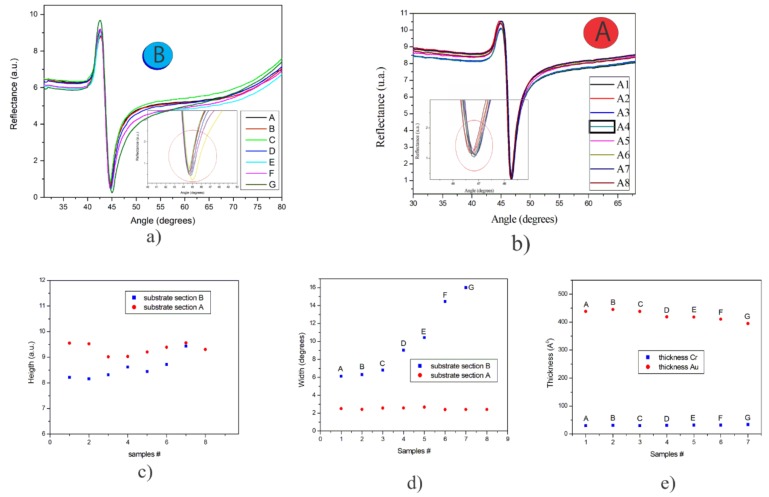
(**a**) SPR curves of the Cr/Au substrates of region B, (**b**) SPR curves of the Cr/Au substrates of region A, (**c**) height of SPR curves of Cr/Au substrates of regions A and B, (**d**) graph of the half-width of the SPR curves of Cr/Au substrates of the regions A and B, (**e**) estimated thickness by experimental/theoretical SPR curve fitting of Cr/Au substrates of region B.

For the uniformity analysis, Cr/Au thin films were evaporated with a rotating mount, which was placed inside the evaporator (see Figure 3a). The substrates were located in seven horizontal lines crossing the eight segments of the mount. The lines were designated as A (top) to line G (bottom); these substrates were called Region B. For the analysis of SPR curves, a substrate of each line was measured (Figure 4a). The use of planetary systems during the evaporation process commonly infers that the substrates would have the same response in the two opposite sides of the system, where the evaporation emitted by the source has a cosenoidal configuration [12]. An SPR analysis was performed for the seven substrates of region B and eight substrates of region A (placed in the upper circle of the planetary system, from substrate A1 to substrate A8).

Figure 4a,b show the experimental SPR curves corresponding to the substrates of region B and region A, respectively. Meanwhile, Figure 4c shows the height of the curves of the two regions, where it can be seen that the substrates of region A show more uniformity than those of region B; even so, the differences in height are minimal, around 1.5 units of reflectance (the units, from 0 to 12, are related to the supply voltage of the photodetector [20]).

Figure 4d shows the half-width of the SPR curves of both A and B regions, denoting that the widths of the SPR curves of region A are very similar, while those of region B show more dispersion ranging from 6 to 16 degrees. The thickness of substrates from region B was estimated through a theoretical/experimental fitting analysis of the SPR curves, taking into account the values of complex refractive index obtained previously in Section 3.1, as shown in Figure 4e.

**Figure 5:**
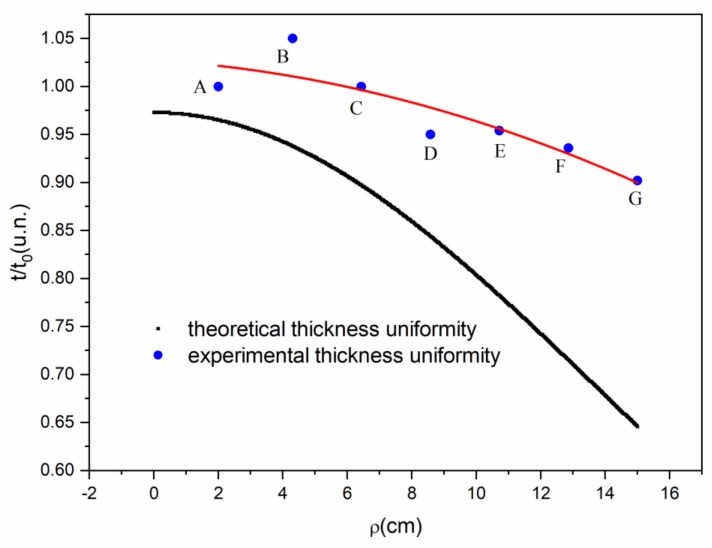
Distribution of the uniformity of the thickness of thin films in evaporation with rotation.

Figure 5 shows a representation of the relative thickness distribution of thin films obtained with a rotation system during the evaporation process. The uniformity of the thickness of each film was estimated from the fitted thickness, adjusted by the square least method, as shown in Figure 4a. Comparing the relative thickness (*t*/*t*_0_) with the uniformity equation, Equation (4) [12], which represents the evaporation of a surface rotating around the average distance R from the source, the deposition parameters of our substrates were: *R* = 15 cm, *ρ* = 22 cm, *h* = 45 cm, and a rotation speed of 6 rpm. Thus, the difference between the substrate on the upper end and the one of the lower end is 20%.

(4) $$t/t_{0}=\frac{\left(1+\frac{R^{2}}{h^{2}}\right)\left[1+\frac{{\left(\rho_{r}\right)}^{2}}{h^{2}}+\frac{R^{2}}{h^{2}}\right]}{{\left[1+\frac{{\left(\rho_{r}\right)}^{2}}{h^{2}}+\frac{R^{2}}{h^{2}}-\left(\frac{2\rho_{r}}{h}\right).\frac{R}{h}\right]}^{3/2}.{\left[1+\frac{{\left(\rho_{r}\right)}^{2}}{h^{2}}+\frac{R^{2}}{h^{2}}+\left(\frac{2\rho_{r}}{h}\right).\frac{R}{h}\right]}^{3/2}}$$

where *t*_0_ is the thickness of the thin film closest to the centre of rotation, *h* is the height of the source to the substrate, *R* is the distance from the source to the centre of rotation, and *ρ* is the distance from the substrate position to the centre of rotation.

## 4. Conclusions

The deposition of thin metal films for use in surface plasmon sensors, particularly in biosensing, necessitates that the parameters of the evaporation process be very well controlled, in order to have excellent repeatability of results. The advantages of performing the evaporation of Cr/Au thin films without rotation are that the substrates close to the thickness monitor will present a more accurate thickness to that required, it is not necessary to run trial-and-error evaporations, and little prior knowledge of the evaporation conditions are required, except the evaporation rate. On the other hand, evaporation with rotation has the advantage of being more efficient than those without rotation because a large number of substrates can be coated in a single run. However, to carry out this method, it is necessary to perform some trial-and-error evaporations, since the thickness of the thin film deposited must be related to the thickness measured by the thickness monitor.
